# Kidney Involvement in PSTPIP1 Associated Inflammatory Diseases (PAID): A Case Report and Review of the Literature

**DOI:** 10.3389/fmed.2021.759092

**Published:** 2021-10-29

**Authors:** Paola Borgia, Riccardo Papa, Matteo D'Alessandro, Roberta Caorsi, Giorgio Piaggio, Andrea Angeletti, Isabella Ceccherini, Gian Marco Ghiggeri, Marco Gattorno

**Affiliations:** ^1^Department of Neuroscience, Rehabilitation, Ophthalmology, Genetics and Maternal and Child Science (DINOGMI), Università degli Studi di Genova, Genoa, Italy; ^2^Autoinflammatory Diseases and Immunodeficiencies Center, IRCCS Istituto Giannina Gaslini, Genoa, Italy; ^3^Division of Nephrology, Dialysis and Transplantation, IRCCS Istituto Giannina Gaslini, Genoa, Italy; ^4^Laboratory of Molecular Nephrology, IRCCS Istituto Giannina Gaslini, Genova, Italy; ^5^Laboratorio di Genetica e Genomica delle Malattie Rare, IRCCS Istituto Giannina Gaslini, Genoa, Italy

**Keywords:** PSTPIP1, PAPA syndrome, PAMI syndrome, kidney, IL-1 inhibitors

## Abstract

Pyogenic arthritis, pyoderma gangrenosum and acne (PAPA) syndrome, and the proline-serine-threonine phosphatase-interacting protein 1 (PSTPIP1)-associated myeloid-related proteinemia inflammatory (PAMI) syndrome are two distinct clinical conditions caused by heterozygous mutations of the *PSTPIP1* gene. While skin and joint involvements are shared by both conditions, PAMI is characterized by hepatosplenomegaly, pancytopenia, and growth failure. Kidney involvement is exceptional in PSTPIP1-mediated disorders. The two missense *PSTPIP1* variants associated with PAMI syndrome are p.E250K and p.E257K. Long-term treatment with interleukin (IL)-1 inhibitors is effective to control inflammatory manifestations and is usually well-tolerated. We report a case of a patient carrying the *PSTPIP1* p.E250K mutation who developed a late-onset kidney involvement despite a long treatment with canakinumab and anakinra. Kidney biopsy showed focal segmental glomerulosclerosis that was treated with tacrolimus (0.1 mg/kg/day in two doses). A literature revision with the aim to assess the proportion and type of kidney involvement in PAMI syndrome revealed that heterogeneous nephropathies may be part of the clinical spectrum. Our study supports the importance of a periodic diagnostic work-up, including kidney laboratory tests and kidney biopsy, in individuals affected with PAMI syndrome. Kidney and liver functions may be impaired regardless of anti-cytokines treatments and additional therapy approaches (i.e., multi-drugs, hematopoietic stem cell transplantation) should be carefully considered.

## Introduction

Proline-serine-threonine phosphatase-interacting protein 1 (PSTPIP1) is a cytoskeleton adaptor protein mainly expressed in hematopoietic cells. Mutations of the *PSTPIP1 gene* were associated with a large group of inflammatory disorders collected under the term PSTPIP1-associated inflammatory diseases (PAID) (1, 2). The clinical spectrum of PAID ranges from a prevalent skin and joint involvement in case of pyogenic arthritis, pyoderma gangrenosum, and acne (pyoderma gangrenosum, acne, pyogenic arthritis, PAPA) syndrome, to more complex phenotypes involving several organs in case of the PSTPIP1-associated myeloid-related proteinemia inflammatory (PAMI) syndrome (3, 4).

The PAMI syndrome is due to the p.E250K and p.E257K missense mutations of the *PSTPIP1* gene. Clinical manifestations may include cytopenia, recurrent infections, vasculitis-associated skin ulcers, hepatomegaly, splenomegaly, lymphadenopathy, and growth failure. High serum calprotectin and zinc concentration is the laboratory hallmark of the disease.

Steroid administration represents the cornerstone of treatment, resulting in effectiveness in about 55% of cases (5). In remaining cases, also the steroid-sparing drugs, such as the anti-cytokines biologics, may result not be completely effective in preventing the long-term complications. Hematopoietic stem cell transplantation has been recently proposed for the management of severe hematological manifestations (i.e., cytopenia) in complicated forms not responsive to conventional anti-cytokine treatments (6).

Here, we report a 22-year-old male previously included in a PAMI cohort (1), receiving chronic treatment with interleukin (IL)-1 inhibitor and presenting kidney involvement resulting in chronic kidney disease. We also present a literature review of this rare complication.

## Case Presentation

A 4-year-old Caucasian boy was referred to our unit for recurrent episodes of asymmetrical polyarthritis at the large joints of the lower limbs (Table 1). Family history was negative. Since he was 6 months, physical examination revealed mild hepatomegaly and splenomegaly. Laboratory tests showed microcytic anemia (hemoglobin 11 g/dL, mean corpuscular volume 70 fL), neutropenia (780 neutrophils/mm^3^), and the bone marrow analysis revealed dyserythropoiesis. Joint aspirations showed sterile but purulent fluid. Partial control of the symptoms was achieved with multiple intra-articular steroid injections.

**Table 1:** Characteristics of the patient.

Age (years*)*	4	8	12	15	19	20	21	22
**Clinical manifestations**								
Sterile pyogenic arthritis	+	–	–	–	–	–	–	–
Pyoderma gangrenosum	–	+	+	–	–	–	–	–
Cystic acne	–	–	+	–	–	+	–	–
Hepato splenomegaly	+	+	+	+	+	+	+	+
Growth failure	–	–	–	+	+	+	+	+
**Laboratory tests**								
Hemoglobin (g/dL)	11	10	11	11	11	11	11	11
While blood cells (number/mmc)	3,000	3,760	3,000	3,260	7,270	10,630	4,900	4,000
C-reactive protein (mg/dL)	ND	ND	5.32	1.64	1.3	11.8	0.86	0.6
Serum amyloid A (mg/L)	ND	ND	73	14.8	42.6	213	3.7	4
Creatininemia (mg/dL)	ND	ND	0.54	0.81	0.79	1.55	1.57	1.7
Proteinuria (g/24 h)	Absent	Absent	Absent	Absent	4.49	4.28	3.18	3.2
**Treatments**								
Intra-articular steroid injections	+	–	–	–	–	–	–	–
Topical steroids and antibiotics	–	+	+	–	–	–	–	–
Oral prednisone	–	–	–	–	–	+	+	–
Canakinumab	–	–	–	+	+	–	+	+
Tacrolimus	–	–	–	–	–	+	–	–
Allopurinol	–	–	–	–	–	+	+	+
ACE inhibitor	–	–	–	–	–	–	+	+

*ACE, angiotensin-converting enzyme; ND, not determined*.

**Figure 1:**
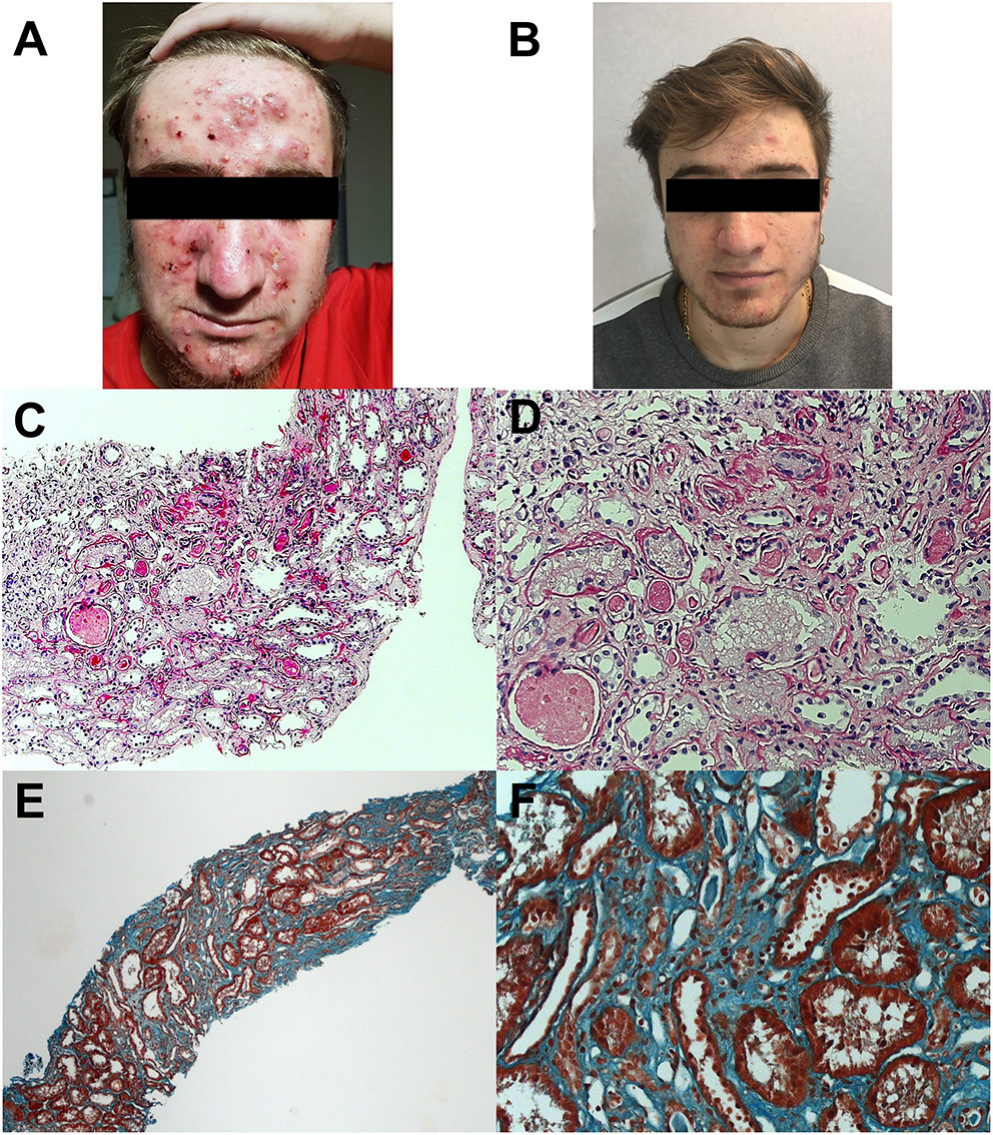
Skin manifestations and kidney histology of the patient. Photos show cystic acne before **(A)** and after **(B)** rechallenge of canakinumab. Histology analysis shows interstitial fibrosis with interstitial infiltration by mononuclear cells and tubular atrophy, classical and with aspects of thyroidization; tubular lumens are dilated and with flaking material; epithelium of some tubules appears vacuolated; segmental hyalinosis of arteriole is also present (**C,D**; periodic acid–Schiff staining; original magnification × 50 and × 200, respectively). Diffuse interstitial fibrosis (**E,F**; trichrome staining; original magnification at × 50 and × 200, respectively).

Since 8 years of age, severe pyoderma gangrenosum started at the periorbital and periungual area [refer to Figure 1B of reference (1)]. Local treatments with antibiotics and steroids were ineffective, and surgical exportation was needed. Due to the very early onset of atypical inflammatory skin and joint involvement and the evidence of high serum levels of zinc and calprotectin (113 μmol/L, normal <50, and 14 μg/mL, normal <0.5, respectively), the Sanger sequencing analysis of the *PSTPIP1* gene was performed, revealing the heterozygous c.748G>A, p.E250K mutation. Therefore, the diagnosis of PAMI syndrome was confirmed.

Since 10 years of age, only the inflammatory skin manifestations persisted: biweekly infusion of human monoclonal anti-tumor necrosis factor (TNF)α antibody adalimumab resulted ineffectively. Since 13 years of age, severe nodulocystic acne has also developed in the face. Oral colchicine was administered but early stopped after 2 weeks due to abdominal pain. IL-1 receptor 1 (IL1R1) antagonist (anakinra) was then attempted, resulting in a prompt clinical improvement of skin lesions. Due to poor patient's compliance, after 2 years, anakinra was replaced with a monthly infusion of the fully human monoclonal anti-IL-1beta antibody canakinumab (7).

At 15 years of age, the weight and height were 48 kg and 147 cm, respectively, revealing a growth failure (10th and <3rd percentile based on Tanner's growth charts). The low serum IGF-1 levels and the abnormal provocative test with arginine confirmed a growth hormone deficiency: the recombinant growth hormone therapy was proposed but refused by the family. After that, the patient performed a blood and urine test every 6 months. At the age of 18, the patient was transferred to a local adult rheumatology unit. Due to administrative issues, the supply of IL-1 monoclonal antibody by the local health system was not regular, leading to transient flares of the skin manifestations, treated with an oral steroid.

At the age of 19 years, the patient was seen for a long-term follow-up visit in our center. Laboratory tests showed new onset of proteinuria in the nephrotic range (4.5 g/daily), microhematuria, and hyperuricemia (9 mg/dL), with normal kidney function (creatininemia 0.8 mg/dl, estimated glomerular filtration rate 129 ml/min/1.73 m^2^). Mild hypoalbuminemia (3,340 mg/dl) and slightly elevated acute phase reactants (C-reactive protein 1.30 mg/dl, serum amyloid A 42.6 mg/L) were also reported. The patient was referred to a local adult nephrologist for competence. Needle kidney biopsy revealed focal segmental glomerulosclerosis (FSGS) without evidence of amyloid deposition. Canakinumab was discontinued, and prednisone (30 mg daily) was started. After 5 months, proteinuria was still in the nephrotic range (4.3 g/daily) with worsening of renal function (creatininemia 1.5 mg/dL, eGFR 63 ml/min/1.73 m^2^). Tacrolimus (maintaining serum range of 4–6 ng/dl) was started, and canakinumab restarted due to worsening of the skin inflammatory manifestations (Figure 1A), resulting in a prompt clinical improvement of skin lesions (Figure 1B). One year later, due to the persistence of proteinuria in the nephrotic range (3.2 g/daily) with stable renal function (creatininemia 1.7 mg/dL, eGFR 56 ml/min/1.73 m^2^), a second kidney biopsy was performed in our center, showing interstitial fibrosis (Figures 1C–F). Thus, tacrolimus was discontinued, prednisone gradually tapered, and he is still receiving canakinumab at the dose of 150 mg monthly.

The acute phase reactants were almost completely normalized (Table 1). Of note, mild anemia and neutropenia persist together with hepatosplenomegaly.

## Discussion

In the context of the clinical spectrum of PSTPIP1-related disorders, renal involvement is reported only in PAMI syndrome. All the English articles found in the PubMed database by querying MEDLINE with the keywords “PSTPIP1” or “PAPA” or “PAMI” were revised. We found 179 articles, among them, only seven articles reported patients with p.E250K or p.E257K mutation, of which four did not report a kidney involvement. In total, only five subjects with the PAMI-associated PSTPIP1 mutations displayed a renal involvement with at least three different phenotypes: glomerular vasculitis in three subjects, tubule-interstitial infiltration, probably related to a systemic inflammatory state, in one subject, and glomerular calprotectin deposition in the remaining one. No evidence of amyloid or immune complex deposition has been reported. Thus, kidney involvement in PAMI seems to present with heterogeneous manifestations and is linked with specific variants of the *PSTPIP1* gene. Of note, 2/6 (33%) patients in Table 2 developed end-stage liver cirrhosis, leading them to liver transplant.

**Table 2:** Reported cases of kidney involvement in PSTPIP1-associated inflammatory diseases.

**Patient number**	**1**	**2**	**3**	**4**	**5**	**6**
Study	Holzinger et al. (1)	Holzinger et al. (1)	Holzinger et al. (1)	Lindwall et al. (8)	Dai et al. (9)	Present case
Gender	Male	Female	Male	Male	Female	Male
Age (years)	35^*^	9	16	25	56	22
Age at onset (years)	6	0	1	4	18	4
Disease duration (years)	29	9	15	19	38	18
*PSTPIP1* p.E250K	Y	Y	Y	Y	Y	Y
**Clinical manifestations**						
Arthritis	Y	N	Y	Y	Y	Y
Pyoderma gangrenosum	Y	N	Y	Y	N	Y
Cystic acne	N	Y	Y	Y	N	Y
Skin ulcers	Y	N	N	Y	N	N
Poor wound healing	Y	N	N	N	Y	Y
Hepatomegaly	Y	Y	Y	Y	Y	Y
Splenomegaly	Y	Y	Y	N	Y	Y
Growth failure	N	Y	Y	N	N	Y
Kidney involvement	Minimal-change glomerulonephritis	IgA nephropathy	Glomerulonephritis	Acute kidney failure	Podocyte effacement and glomerular calprotectin dense deposits	Focal segmental glomerulosclerosis
Others	Liver cirrhosis, post liver transplant complications	Mild lymphadenopathy, arthralgia, gastrostomy tube feeding, familiarity for early gout	Aseptic necrosis of femoral head	Osteomyelitis, acute cholecystitis, sepsis, colitis, cellulitis, acute respiratory failure, epistaxis, joint and skin laxity, familiarity for psoriatic arthritis	Recurrent pneumonia, lymphadenopathy, macronodular cirrhosis with mild portal hypertension	Growth hormone deficiency
**Laboratory tests**						
Anemia	Y	Y	Y	Y	Y	Y
Neutropenia	Y	Y	Y	Y	Y	Y
Others	N	Thrombocytopenia, *MEFV* p.E148Q carrier	N	N	Von Willebrand's factor deficiency, high agammaglobulinemia	Dyserythropoiesis at the bone marrow biopsy
**Treatments**						
Steroid-sparing drugs (duration; clinical response)	Anakinra (2 months; partial), infliximab (3 months; partial)	Cyclosporin (ND; partial), colchicine (NT) anakinra (ND; complete)	Cyclosporin (ND; partial), mycophenolate mofetil (ND; none), rituximab (NT), tocilizumab (ND; partial)	Infliximab (ND; none)	Sulfasalazine (ND; none), methotrexate (ND; none), colchicine (ND; complete)	Colchicine (NT), adalimumab (3 months; none), anakinra (ND; complete), canakinumab (ND; complete), tacrolimus (ND; partial)

* *Age of death; ND, not determined; NT, not tolerated; PSTPIP1, proline-serine-threonine phosphatase-interacting protein 1*.

**Figure 2:**
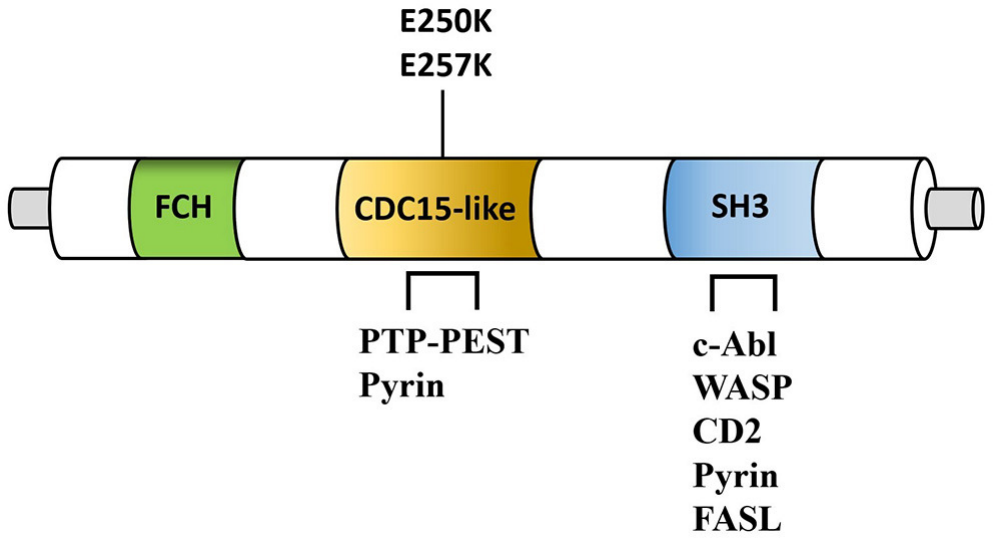
Simplified structure of PSTPIP1 protein. Fes/CIP4 homology domain (FCH), CDC15-like adaptor protein (CDC15-like), SRC Homology 3 Domain (SH3) are shown. Note the position of the PAMI-associated mutations (E250K and E257K). PSTPIP1 interacting proteins include the protein-tyrosine phosphatase (PTP-PEST), pyrin, the Wiskott–Aldrich syndrome protein (WASP), the c-Abl kinase, CD2, and the Fas ligand (FASL). PSTPIP1, proline-serine-threonine phosphatase-interacting protein 1.

The triad of vasculitis, cytopenia, and lymphoproliferation described in PAMI can be related to specific ligands of the PSTPIP1 protein. In fact, PSTPIP1 is not expressed primitively in the kidney, whereas it is highly expressed in hematopoietic cells. The protein is able to bind with immune-related proteins, like the cytosolic protein tyrosine phosphatase (PTP-PEST), the Wiskott–Aldrich syndrome protein (WASP), the c-Abl kinase (ABL), the CD2, and the Fas ligand, probably with the aim to counteract the cytotoxic cell functioning (10) (Figure 2).

Mutations associated with PAID decrease the binding activity of the protein to PTP-PEST (11), suggesting that gain and loss of function should be considered. Furthermore, despite T cells do not show a prevalent involvement in PAID lesions, the mild lymphoproliferation described in some patients with PAMI may be related to a T-cell activation defect caused by abnormal CD2 binding activity or absence of the Fas ligand, may be due to its sequestration into lysosomes. Moreover, WASP and ABL are actin-related proteins and the cytopenia and vasculitis of PAMI patients may be secondary to their dysfunction, as reported in patients with actin remodeling defects (12, 13). Thus, PAMI syndrome represents the most severe form of the PAID spectrum, and its clinical manifestations may be secondary also to actin remodeling defect and lymphocytes hyperactivation, other than the high IL-1 production as for others PAID.

As reported, kidney biopsy was negative for amyloid deposition (14), in line with previous reports (Table 2), and the histological findings do not support calprotectin deposition as recently described (9). Therefore, other pathogenic mechanisms causing kidney lesions should be investigated. In the first kidney biopsy, a diagnosis of FSGS was reported. The pathogenic mechanism of FSGS remains still poorly understood (15). Recent findings demonstrated the role of the IL-1 pathway as a possible pathogenic mechanism in proteinuric disease, supporting therefore the administration of treatments blocking the IL-1β/IL-1R1 signaling to delay the development of sclerotic lesions (16). In the second kidney biopsy, performed despite stable renal function and proteinuria, a diffused tubule-interstitial fibrosis was revealed, probably due to tacrolimus administration, thus discontinued. Notably, our patient developed proteinuria after irregular administration of IL-1 inhibitors: scarce adherence to IL-1 receptor antagonist (anakinra) was admitted, as above reported and, due to local administrative issues, the supplying of canakinumab was rather inconstant. At this stage, it is difficult to determine the real IL-1 dependence of the renal manifestation.

As reported in Table 2, in patient 4, colchicine was effective in reversing proteinuria. A multi-drug approach may control various manifestations of PAMI by silencing the concomitant defects of different components of the immune system. Furthermore, it remains a matter of speculation that the kidney involvement of other pyrin-related autoinflammatory disorders may not be due only to amyloid deposition. More studies are required to investigate the mechanisms of renal involvement and the possible role of anti-IL drugs against these manifestations.

In conclusion, PAMI syndrome is a rare inflammatory disorder and the most severe phenotype among PAID, characterized by alterations of various immune system agents. The severe kidney involvement of our patient is, according to our best knowledge, the first subjected to a documented histological modification after anti-IL-1 treatment.

## Data Availability Statement

The original contributions presented in the study are included in the article/supplementary material, further inquiries can be directed to the corresponding author/s.

## Ethics Statement

The studies involving human participants were reviewed and approved by Ethics Committee Genoa, Italy. Written informed consent to participate in this study was provided by the participants' legal guardian/next of kin. Written informed consent was obtained from the individual(s), and minor(s)' legal guardian/next of kin, for the publication of any potentially identifiable images or data included in this article.

## Author Contributions

PB, RP, AA, GG, and MG conceptualized the manuscript. PB and RP conducted the literature review and drafted the manuscript. MD'A, RC, and GP were involved in the clinical care of the patient. IC performed the genetic analysis. All authors read and approved the final version of the submitted manuscript.

## Funding

The study was supported with public funds granted by the Italian Ministry of Health.

## Conflict of Interest

The authors declare that the research was conducted in the absence of any commercial or financial relationships that could be construed as a potential conflict of interest.

## Publisher's Note

All claims expressed in this article are solely those of the authors and do not necessarily represent those of their affiliated organizations, or those of the publisher, the editors and the reviewers. Any product that may be evaluated in this article, or claim that may be made by its manufacturer, is not guaranteed or endorsed by the publisher.
